# Organic acid-mediated phosphorus mobilization in black soils: differential effects of maize root exudates on alfisols and mollisols in Northeast China

**DOI:** 10.1371/journal.pone.0333230

**Published:** 2025-09-24

**Authors:** Wenzhi Zhao, Yuan Zhang, Xu Xie, He Li, Jintao Zhang

**Affiliations:** 1 Center for Harbin Natural Resources Comprehensive Survey, China Geological Survey, Harbin, P. R. China; 2 Observation and Research Station of Earth Critical Zone in Black Soil, Ministry of Natural Resources, Harbin, P. R. China; University of Minnesota, UNITED STATES OF AMERICA

## Abstract

Soil phosphorus exists in various forms, but only minimal soluble phosphorus is bioavailable. While phosphate fertilizers address deficiency, low efficiency leads to insoluble phosphate accumulation, wasting resources. Enhancing phosphorus availability by converting non-available forms is a critical research priority. Under phosphorus-deficient conditions, plants enhance soil phosphorus availability by secreting organic acids. This study aims to explore phosphorus activation in Alfisol and Mollisol soils through maize root-secreted organic acids. Semi-hydroponic maize cultivation under low/medium/high phosphorus levels and sterilized soil (Alfisol/Mollisol) incubation with root-secreted organic acids (0.5–2%). Exudates analyzed via UHPLC-MS/MS; soil phosphorus dynamics monitored over 60 days. Under phosphorus limitation, maize root exudates showed marked increases in malic, citric, tartaric, and trans-aconitic acids. Soil incubation revealed peak phosphorus release during 0–20 d, with Alfisol exhibiting 95.8% (tartaric), 91.1% (citric), 81.8% (malic), and 67.7% (trans-aconitic) increases in available phosphorus, while Mollisol showed 94.0%, 119.6%, 83.1%, and 75.2% increments, respectively. Tartaric and citric acids outperformed others, boosting H_2_O-P and NaHCO_3_-P_i_, whereas malic and trans-aconitic acids mainly elevated NaHCO_3_-P_i_. Fractionation analysis highlighted distinct mechanisms: citric acid primarily mobilizes HCl-P; malic acid mainly targets NaOH-P_i_ and HCl-P; tartaric acid mobilizes NaOH-P_o_, HCl-P, and NaOH-P_i_; while trans-aconitic acid influences NaOH-P_o_, HCl-P, and Res-P. These findings demonstrate acid-specific phosphorus pool mobilization, identifying tartaric and citric acids as most effective, supporting optimized phosphorus management strategies in black soils.

## 1  Introduction

Phosphorus (P), a critical yet frequently limiting nutrient for plant productivity and ecosystem functioning in terrestrial environments, exhibits substantial variation in bioavailability due to its complex speciation in soils [1,2]. In northeast China’s black soil regions, while total phosphorus (TP) reserves are abundant, low bioavailability persists due to mineral adsorption, organic complexation, and land management practices [3]. Conventional phosphate fertilization, though widely adopted to address P deficiencies and enhance crop yields, suffers from limited efficiency as 70–90% of applied P becomes immobilized in insoluble calcium-, iron-, and aluminum-phosphate forms, exacerbating resource waste [4,5]. Furthermore, unutilized P migrates via leaching and runoff, posing risks of eutrophication in aquatic systems and groundwater contamination [6]. Given that phosphate rock, the primary source of P fertilizers, is a finite, non-renewable resource facing escalating extraction costs and diminishing reserves, innovative strategies to enhance soil P availability through speciation conversion are urgently required [7].

The challenge of phosphorus inefficiency is particularly acute in the globally vital black soil regions, such as Northeast China’s “breadbasket”. These deep, carbon-rich soils are among the world’s most productive but face significant degradation threats, including organic matter decline and nutrient imbalances, which exacerbate phosphorus fixation and reduce fertilizer efficacy [8,9]. Enhancing P bioavailability in these critical agroecosystems is thus not merely a local concern but a cornerstone of global food security, given their outsized contribution to staple grain production. Organic acid-mediated phosphorus mobilization, leveraging a naturally evolved plant strategy, represents a fundamentally sustainable pathway with global relevance [10–13]. By harnessing inherent root processes to unlock fixed soil P reserves, this approach reduces dependence on finite, geopolitically concentrated rock phosphate resources [14–17].

Critically, optimizing P bioavailability through organic acids in black soils offers significant co-benefits for climate change mitigation. By improving the efficiency of existing soil P reserves and reducing fertilizer demand, organic acid-mediated strategies can lower the carbon footprint of crop production in these regions [18]. Furthermore, reducing P runoff protects water quality and mitigates a potent secondary greenhouse gas (GHG) source: eutrophic aquatic systems. The vast scale of agriculture on the Northeast China black soil belt means that widespread adoption of such P-efficiency strategies could yield substantial regional GHG reductions [19,20]. For black soils specifically, minimizing tillage disturbance for fertilizer incorporation–often replaced by targeted organic acid approaches–also helps preserve soil organic carbon, further contributing to climate goals [21,22].

Under phosphorus deficiency, plants enhance rhizospheric phosphorus (P) availability through root-secreted organic acids, which mobilize fixed P via ligand exchange and competitive adsorption [23–29]. However, critical knowledge gaps persist. Existing studies predominantly quantify total P mobilization but lack resolution on how specific organic acids target distinct P fractions across divergent soil type [30–32]. Furthermore, the differential responsiveness of Alfisols and Mollisols to organic acid-mediated P activation remains poorly characterized, despite their contrasting mineralogy and organic matter content. Agronomically, while maize is a key crop in Northeast China, its root exudate profile under P limitation-and the subsequent impact on regional soils-has not been systematically linked to P fraction dynamics [33,34]. Consequently, current strategies for optimizing P bioavailability remain generic, with limited capacity to tailor interventions based on soil-specific P speciation or crop exudate signatures.

To bridge these gaps, this study integrates root exudation chemistry with soil P fractionation kinetics. Using semi-hydroponic maize cultivation and sterile soil incubation, we investigated: 1) genotype-specific organic acid secretion under P limitation; 2) differential activation pathways of four key organic acids (citric, malic, tartaric, trans-aconitic) on Alfisol versus Mollisol P pools; and 3) time-dependent redistribution of Hedley P fraction to establish acid- and soil-specific mobilization mechanisms.

## 2 Materials and methods

### 2.1 Experimental design I: Semi-hydroponic maize cultivation for root exudate collection

#### (1) Semi-hydroponic experiment of maize.

Following surface sterilization with 10% H₂O₂ for 10 minutes, maize seeds of three varieties (SY-1, ZD-958, XY-335) were thoroughly rinsed with distilled water, soaked in warm water for 10 hours, and germinated in a semi-hydroponic system under controlled humidity maintained by daily irrigation with distilled water. Upon emergence of the first leaf, seedlings were subjected to nutrient solutions with three phosphorus (P) levels: low (LP, 1 μM P), medium (MP, 100 μM P), and high (HP, 250 μM P), supplied via KH₂PO₄. Potassium concentrations between LP and MP treatments were balanced using KCl. Nutrient solutions (initial pH 6.0) were refreshed every 3 days, with pH readjusted to 6.0 using 1 M HCl or NaOH during each replacement to ensure consistent growth conditions.

#### (2) Collection and analysis of root-secreted organic acids.

Root exudates were collected from 18-day-old maize plants using a standardized protocol: Roots were gently rinsed 3–5 times with distilled water during morning hours (10:00 AM), then immersed in 0.5 mM CaCl₂ solution for 4 h under microbial inhibition with 0.01% thymol [35]. The collected solution underwent sequential filtration through qualitative filter paper (removing particulate residues) followed by a 0.22 μm nylon membrane. Processed exudates were immediately concentrated, flash-frozen at −80°C, and stored at −20°C until analysis. Organic acid profiles were quantitatively determined using ultra-high-performance liquid chromatography coupled with tandem mass spectrometry (UHPLC-MS/MS) with external calibration standards [36].

### 2.2 Experimental Design II: Sterile soil incubation for phosphorus activation

Soil incubation period and organic acid concentration selection were based on preliminary kinetic studies and established literature on organic acid-mediated P release in black soils. Previous research indicates that maximum P solubilization typically occurs within 2–4 weeks of organic acid application, followed by potential re-adsorption or stabilization. A 60-day incubation period was chosen to capture the complete dynamic trajectory of P release, stabilization, and potential decline, ensuring observation of both short-term solubilization peaks (expected within 0–20 d) and longer-term equilibrium states [37,38]. Similarly, the applied organic acid concentrations (0.5–2% w/w) were selected to reflect: 1) the range of physiologically relevant concentrations observed in maize rhizosphere microsites under P stress, where localized concentrations can significantly exceed bulk soil levels; and 2) concentrations demonstrated in prior incubation studies to effectively mobilize recalcitrant P pools in Alfisols and Mollisols without inducing excessive artificial acidification beyond field-relevant conditions [39].

Soil samples (0–20 cm depth) were collected from two long-term maize fields in Heilongjiang Province, China: brown soil (Alfisol) from Heihe field observation station (127°26′56″ E, 50°15′47″ N) and black soil (Mollisol) from Hulan field observation station (126°39′49″ E, 45°58′26″ N). Soils were air-dried, sterilized by gamma irradiation (25 kGy), and sieved through a 100-mesh stainless steel screen (0.15 mm). Triplicate 300 g aliquots were transferred to sterile 500 mL polypropylene bottles and incubated at 25°C with gravimetric moisture maintained at 0.3 g·g ⁻ ¹ (30% water-holding capacity). After a 7-day acclimation phase, soils underwent sterilization validation through microbial plate assays to confirm sterility. Heihe and Hulan field observation station permitted the work, field site access, and soil sampling in cultivated areas.

Root-secreted organic acids (0.5%, 1%, 1.5%, 2% w/w) were aseptically applied as phosphorus activators, with untreated controls (CK) receiving equivalent sterile water. Treatments were homogenized under laminar airflow to prevent contamination. Soil sampling was conducted at eight intervals (0, 5, 10, 20, 30, 40, 50, 60 days post-treatment), with triplicate samples immediately frozen at −80°C for subsequent physicochemical analyses.

### 2.3 Soil physicochemical characterization

Total phosphorus (TP) content was determined via inductively coupled plasma optical emission spectroscopy, while available phosphorus (AP) was extracted with NH₄Cl-HCl and measured by molybdenum-antimony anti-spectrophotometry. Phosphorus speciation analysis using the Hedley sequential fractionation method revealed distinct partitioning patterns between the Alfisol and Mollisol soils..All analytical determinations were performed in triplicate, with results expressed as mean ± standard error (SE). Key soil properties are summarized in **Table 1**. Both soil types exhibit acidic conditions (pH < 7), with AP representing 3.59% and 6.34% of TP in the Alfisol and Mollisol soils, respectively, highlighting limited phosphorus bioavailability in both pedological systems.

**Table 1:** Basic characteristics of soils in the study area.

Soil types	pH	H_2_O-P_i_ (mg kg^-1^)	NaHCO_3_-P_i_ (mg kg^-1^)	NaHCO_3_-P_o_ (mg kg^-1^)	NaOH-P_i_ (mg kg^-1^)	NaOH-P_o_ (mg kg^-1^)	HCl-P (mg kg^-1^)	Res-P (mg kg^-1^)	TP (mg kg^-1^)	AP (mg kg^-1^)
Alfisol	6.7 ± 0.2	5.2 ± 0.3	12.3 ± 0.4	40.4 ± 2.2	71.0 ± 3.7	223 ± 11.2	96.9 ± 4.1	121 ± 4.3	551 ± 21.1	19.8 ± 2.7
Mollisol	5.7 ± 0.2	10.5 ± 0.4	25.4 ± 1.7	70.1 ± 3.5	107 ± 4.6	210 ± 9.8	122 ± 4.5	127 ± 5.1	642 ± 33.0	40.7 ± 3.6

Data are represented as mean±SE (Standard Error, n = 3).

Labile phosphorus pools, including H₂O-extractable inorganic P (H₂O-Pi: 0.91% vs. 1.56%) and NaHCO₃-extractable fractions (inorganic NaHCO₃-P_i_: 2.15% vs. 3.78%; organic NaHCO₃-P_o_: 7.09% vs. 10.43%), collectively constituted the bioavailable P reservoirs, with the Mollisol exhibiting higher labile P proportions. Moderately labile NaOH-extractable organic P (NaOH-P_o_) dominated both soils, representing 39.19% (Alfisol) and 31.25% (Mollisol) of total P. Stable P fractions (HCl-P + Res-P) accounted for 18–20% of total P in both pedons, demonstrating comparable long-term P sequestration capacities despite divergent pedogenic processes.

### 2.4 Statistical analysis

Statistical analyses were performed using SPSS 18.0 (IBM Corp., Armonk, NY, USA) and Canoco 4.5 (Microcomputer Power, Ithaca, NY, USA). Redundancy analysis (RDA), a constrained ordination method, was conducted to quantify multivariate relationships between P speciation (Hedley fractions) and environmental variables. RDA was selected over alternative multivariate methods due to its ability to explicitly model the relationship between response variables (P fractions) and explanatory environmental variables (soil physicochemical properties). As a constrained ordination technique, RDA directly tests hypotheses regarding the influence of predefined environmental factors on multivariate response data, aligning with our objective to quantify drivers of phosphorus fraction dynamics under organic acid treatments.

Prior to analysis, data validation procedures were implemented: 1) Potential outliers were identified via visual inspection of boxplots and scatterplots, with data points exceeding ±3 standard deviations from the mean flagged; 2) No outliers required exclusion as all values fell within biologically plausible ranges; 3) Multicollinearity among predictors was assessed via variance inflation factors (VIF), with all variables retaining VIF < 5, well below the critical threshold of 10.

## 3 Results

### 3.1 Changes of organic acids secreted by maize roots under phosphorus restriction

**Fig 1:**
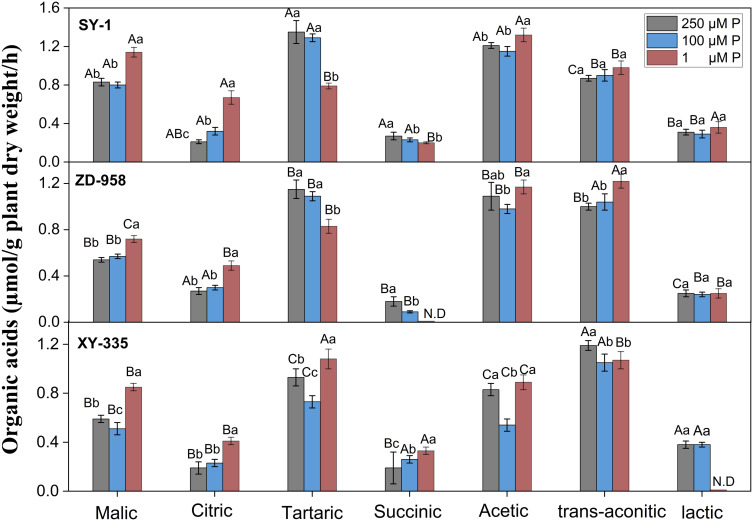
Effects of varying phosphorus supplies on the rhizosphere organic acid concentrations of three maize varieties. Different lowercase letters indicate significant differences among phosphorus treatments for the same organic acid; different uppercase letters indicate significant differences among maize varieties for the same organic acid.

Under varying phosphorus regimes, three maize varieties (SY-1, ZD-958, XY-335) demonstrated genotype-specific organic acid secretion patterns in root exudates (**Fig 1**). Acetic, malic, tartaric, and trans-aconitic acids emerged as dominant components across all varieties, while succinic and lactic acids showed minimal secretion. Low phosphorus (LP, 1 μM P) treatment markedly stimulated citric and malic acid production in all genotypes, with SY-1 exhibiting superior adaptive responses through 34.1–63.4% higher secretion of these acids compared to ZD-958 and XY-335.

Distinct varietal responses emerged in tartaric and trans-aconitic acid dynamics under phosphorus limitation. SY-1 and ZD-958 showed progressive decreases in tartaric acid secretion under LP stress, contrasting with XY-335’s 16.1–47.9% increase. Conversely, trans-aconitic acid secretion exhibited inverse patterns: LP treatment enhanced its production in SY-1 and ZD-958 by 3.4–22.0%, while suppressing secretion in XY-335 by 11.2–13.3%. These differential regulatory mechanisms highlight genotype-dependent strategies for phosphorus acquisition, with SY-1 demonstrating particularly effective metabolic adjustments through coordinated upregulation of key organic acids under nutrient deprivation.

These genotype-specific exudation profiles have direct implications for maize breeding programs. SY-1’s superior capacity to upregulate citric and malic acid secretion under P limitation positions it as a prime candidate for developing P-efficient genotypes. Incorporating such root exudate traits into breeding schemes could reduce fertilizer dependency in black soil regions, aligning with sustainable intensification goals.

### 3.2 Influence of different treatment levels on soil AP

Soil activation experiments were conducted using four maize root-secreted organic acids–malic acid, citric acid, tartaric acid, and trans-aconitic acid–applied at five concentrations (0%, 0.5%, 1%, 1.5%, 2% w/w). Phosphorus fractionation dynamics were monitored over a 60-day incubation period, with temporal sampling at 5, 10, 20, 30, 40, 50, and 60 days post-application.

**Fig 2:**
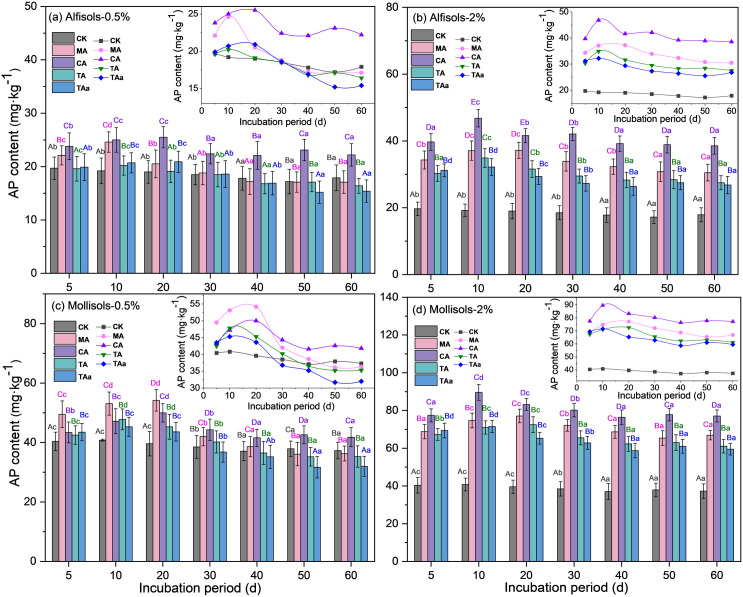
Changes in the AP content at various time points in soils amended with organic acids over the 60 d incubation period. Different lowercase letters indicate significant differences among incubation times for the same organic acid; different uppercase letters indicate significant differences among organic acids at the same incubation time. Panels: AP variation in organic acid-amended soils across 60 d. AP: available P.

The activation of AP in both soils demonstrated concentration-dependent variations in response to organic acid applications, as illustrated in **Fig 2**. Higher organic acid concentrations (0.5–2%) consistently enhanced AP mobilization, with maximal activation observed at the 2% treatment level. After application, AP concentrations increased sharply during the first 10–15 days, followed by a gradual decline and stabilization. This biphasic response–rapid solubilization followed by stabilization–was consistent across all four organic acids, with activation magnitudes directly proportional to applied concentrations.

In Alfisol (**Fig 2a**, **2b**), all organic acids significantly increased AP concentrations at equivalent application rates (0.5–2% w/w), with efficacy ranking: citric acid > malic acid > tartaric acid > trans-aconitic acid. Citric acid induced the strongest activation, elevating AP by 21.1–30.2% (0.5%), 31.0–49.5% (1%), 64.0–91.1% (1.5%), and 101.5–143.8% (2%). Malic acid showed moderate effects, ranging from minimal change up to 28.1% (0.5%) to 70.4–95.8% (2%), while tartaric and trans-aconitic acids had weaker initial responses at <1% concentration but still achieved 53.6–81.8% and 48.3–67.7% increases at 2%, respectively.

In Mollisol (**Fig 2c**, **2d**), all acids enhanced AP across sequential incubation periods at identical concentrations. Citric acid generated the highest increments (7.4–26.3% to 91.6–119.6%), followed by malic acid (up to 17.2% initially, rising to 83.1%), tartaric acid (5.0–59.8% to 70.3–94.0%), and trans-aconitic acid (effects up to 11.0% initially, reaching 75.2%). Notably, malic acid outperformed citric acid at low concentrations (<0.5% w/w) during early incubation (≤20 days), but citric acid surpassed all others above 1% concentration, peaking at 119.6% elevation.

While the efficacy hierarchy remained consistent across both Alfisol and Mollisol, the magnitude of AP activation varied significantly. Citric acid induced substantially greater AP elevation in Alfisol than in Mollisol. Similarly, malic acid’s performance was more pronounced in Alfisol versus Mollisol. Trans-aconitic acid consistently exhibited the weakest effects in both soils, though its lowest concentration (0.5%) triggered near-neutral responses in Alfisol compared to slightly positive effects in Mollisol. The anomaly of malic acid surpassing citric acid at <0.5% w/w during early incubation was exclusive to Mollisol, highlighting soil-specific ligand interactions at trace concentrations.

The observed concentration threshold for net P mobilization has practical significance for amendment formulations. Field applications should exceed this critical concentration to avoid unintended P immobilization, particularly in Alfisols where adsorption dominance was more pronounced. Precision delivery systems could optimize acid efficacy while minimizing application rates.

The cultivation trials revealed that elevated concentrations of organic acids (2% w/w) elicited the most pronounced modifications in phosphorus fractionation profiles across both soil types. Consequently, subsequent experiments were conducted using elevated concentrations of activators.

### 3.3 Differential responses of soil P Pools to high-dose organic acid amendments

**Fig 3:**
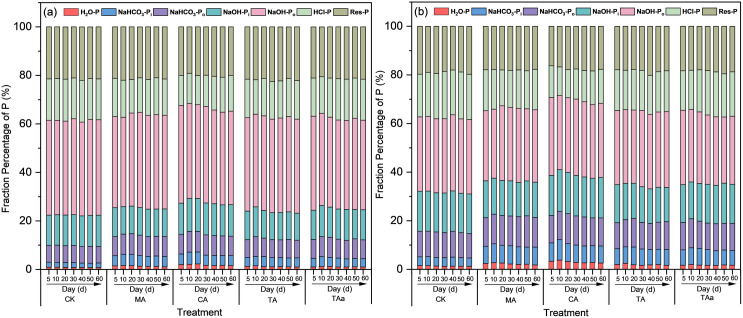
Average percentage of different P fractions at various time points in soils amended with organic acids (organic acids applied at 2% by weight in the incubated soil) over the 60 d incubation period for (a) Alfisol, (b) Mollisol.

The effects of citric acid, tartaric acid, malic acid and trans-aconitic acid at a concentration of 2% on soil P fractions in two types of soil were depicted in **Fig 3**.

In the Alfisol soil (**Fig 3a**), sequential extraction analysis revealed that citric acid amendment significantly enhanced labile phosphorus fractions, with H_2_O-P and NaHCO_3_-P_i_ contents increasing to 2.28% and 4.87% respectively by Day 20, showing a marked elevation compared to the CK values (0.84% and 2.20%). Both fractions exhibited an initial increase from Day 0 to Day 20 followed by a gradual decline, yet maintained consistently higher concentrations than CK throughout the experiment. The organic phosphorus component NaHCO_3_-P_o_ showed a transient increase during early incubation before decreasing to stabilization, while NaOH-P_o_ demonstrated a progressive reduction over time, suggesting stimulated mineralization processes [27]. The HCl-P fraction decreased continuously with prolonged incubation, contributing to the observed enhancement in labile P pools. Notably, the NaOH-P_i_ and residual-P fractions remained stable without significant variations during the 20-day experimental period.

Tartaric acid application induced comparable dynamics to citric acid treatment, with H_2_O-P, NaHCO_3_-P_i_, and NaHCO_3_-P_o_ exhibiting similar temporal patterns. The NaOH-P_i_ fraction showed a gradual decline throughout the incubation period, while NaOH-P_o_ and HCl-P concentrations displayed a distinct trajectory: an initial reduction, followed by partial recovery, and eventual stabilization at values below those observed in the CK soil. Notably, the Res-P pool remained stable without significant variations despite prolonged cultivation.

Malic acid treatment significantly enhanced the NaHCO_3_-P_i_ fraction, peaking at 4.23%, while H_2_O-P and NaHCO_3_-P_o_ exhibited a transient increase followed by stabilization with prolonged incubation. Concurrently, NaOH-P_i_ and HCl-P displayed dynamic trends characterized by an initial decline, subsequent recovery, and eventual stabilization at levels below those in the CK soil. In contrast, NaOH-P_o_ and Res-P remained stable without significant variations throughout the experimental period, highlighting the selective mobilization of specific phosphorus pools under organic acid treatment.

Sequential extraction analysis revealed distinct phosphorus mobilization patterns under trans-aconitic acid treatment. The NaHCO_3_-P_i_ fraction exhibited significant activation, peaking at 4.06%, while H_2_O-P and NaHCO_3_-P_o_ showed marginal increases (slightly above CK levels) with transient fluctuations before stabilization. The HCl-P fraction displayed a biphasic response–initial reduction followed by recovery and stabilization–though remaining below CK values. In contrast, NaOH-P_o_ exhibited a progressive decrease with prolonged incubation, while NaOH-P_i_ and Res-P remained stable without significant variations. These trends underscore the selective activation and redistribution of specific phosphorus pools induced by trans-aconitic acid.

In the Mollisol soil (**Fig 3b**), Sequential extraction analysis demonstrated pronounced phosphorus fraction redistribution in Mollisol under organic acid treatments. Citric acid significantly enhanced labile P pools, elevating H_2_O-P and NaHCO_3_-P_i_ concentrations, while NaHCO_3_-P_o_ and NaOH-P_i_ exhibited an initial rise followed by stabilization at levels exceeding CK. Notably, HCl-P decreased substantially, reaching 11.55% by Day 20 compared to 18.65% in CK. Tartaric, malic, and trans-aconitic acids induced similar trends for H_2_O-P, NaHCO_3_-P_i_, and NaHCO_3_-P_o_ as citric acid, though with NaOH-P_i_ consistently lower than CK. Mechanistic divergence emerged in P activation pathways: citric, tartaric, and malic acids primarily mobilized labile P through HCl-P depletion, whereas trans-aconitic acid preferentially reduced NaOH-P_o_ (from 12.4% to 9.2% by Day 20) to enhance labile P pools. Residual phosphorus remained stable across treatments, confirming selective chemical mobilization of specific fractions.

Comparative analysis revealed differential phosphorus mobilization capacities among organic acids across contrasting soil matrices, with Mollisol exhibiting greater responsiveness than Alfisol in labile phosphorus pool redistribution. Citric acid and tartaric acid treatments demonstrated superior efficacy in activating labile P fractions relative to malic acid and trans-aconitic acid, aligning with their distinct chemical activation pathways. This hierarchy of organic acid performance underscores soil-specific interactions in phosphorus fraction dynamics, where Mollisol’s enhanced reactivity amplified treatment effects compared to Alfisol’s more conservative response.

## 4 Discussion

**Fig 4:**
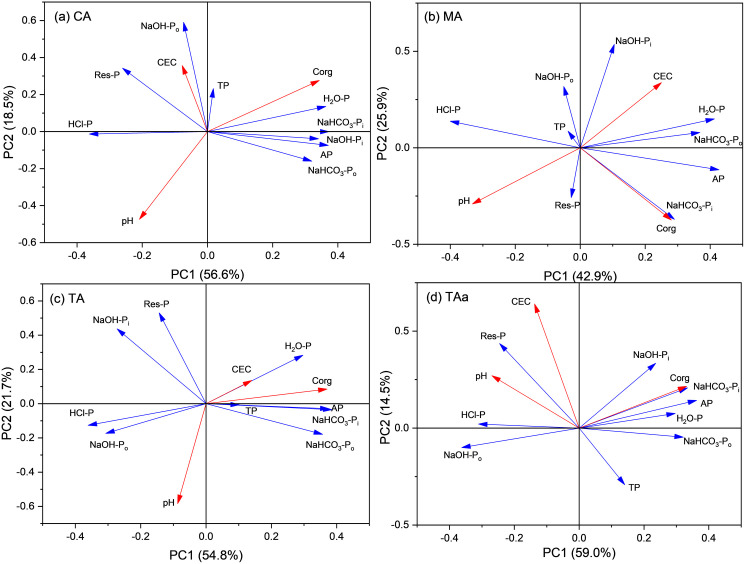
Results of RDA for P fractions and soil physicochemical properties in 2% level treatments.

Redundancy analysis (RDA) revealed significant ordination patterns between soil P fractions and physicochemical properties under organic acid treatments (**Fig 4**).

In citric acid-treated soils (**Fig 4a**), pH exhibited a significant negative correlation with H_2_O-P, NaHCO_3_-P_i_, and AP, but a positive correlation with HCl-P. AP showed strong positive correlations with H_2_O-P, NaHCO_3_-P_i_, NaHCO_3_-P_o_, and NaOH-P_i_, and negative correlations with HCl-P, Res-P, and NaOH-P_o_, indicating its primary derivation from labile and moderately labile P fractions (H_2_O-P, NaHCO_3_-P_i_, NaHCO_3_-P_o_, NaOH-P_i_). The significant negative correlation between HCl-P and AP, as well as with other labile P components, identifies HCl-P as the principal source of mobilized phosphorus. This aligns with observed HCl-P reduction and concurrent AP elevation. While citric acid-induced pH decline correlated strongly with HCl-P dissolution, sustained AP elevation post-pH recovery (Day > 20) suggests complementary activation mechanisms. Ligand exchange between carboxylic anions and HPO_4_^2-^/H_2_PO_4_^-^ likely contributed to P release beyond initial acidification effects, as evidenced by persistent AP levels exceeding controls despite pH stabilization.

Malic acid treatment (**Fig 4b**) induced concurrent reductions in NaOH-P_i_ and HCl-P across both soils, signifying decreased Fe/Al/Ca-associated inorganic phosphorus (P_i_). Comparative analysis revealed lower phosphorus activation efficacy relative to citric acid. Significant negative correlations emerged between pH and H_2_O-P, NaHCO_3_-P_i_, AP, and NaHCO_3_-P_o_, while pH showed a positive correlation with HCl-P. HCl-P exhibited strong negative correlations with AP and labile P fractions (H_2_O-P, NaHCO_3_-P_i_, NaHCO_3_-P_o_), mirroring the mobilization patterns observed under citric acid treatment. These parallel correlation dynamics suggest shared activation mechanisms between the two organic acids, likely involving proton-mediated dissolution of mineral-bound P and ligand exchange processes.

Tartaric acid treatment (**Fig 4c**) demonstrated distinct phosphorus mobilization dynamics, with pH exhibiting significant negative correlations with H_2_O-P, NaHCO_3_-P_i_, and AP, and positive correlations with NaOH-P_o_ and HCl-P. Strong negative correlations emerged between NaOH-P_i_, NaOH-P_o_, HCl-P, and labile P fractions (AP, NaHCO_3_-P_i_, H_2_O-P, NaHCO_3_-P_o_), identifying these stable P pools as contributors to labile P mobilization. HCl-P displayed the strongest inverse relationship with AP, indicating its dominant role in P activation. Concurrently, the progressive decline in NaOH-P_o_ concentration (from 12.4% to 9.2% by Day 20) suggests partial mineralization of organically bound phosphorus facilitated by tartaric acid. These patterns highlight a dual activation mechanism combining mineral-bound P dissolution (HCl-P depletion) and organic P transformation (NaOH-P_o_ reduction), distinct from other organic acid treatments.

Trans-aconitic acid treatment (**Fig 4d**) revealed distinct phosphorus mobilization dynamics, with pH demonstrating significant negative correlations with H_2_O-P, NaHCO_3_-P_i_, and AP, contrasting with positive associations observed for NaOH-P_o_, HCl-P, and Res-P. Strong inverse relationships emerged between NaOH-P_o_, HCl-P, Res-P, and labile P fractions (AP, H_2_O-P, NaHCO_3_-P_i_, NaHCO_3_-P_o_), identifying these stable pools as contributors to phosphorus release. NaOH-P_o_ exhibited the most pronounced negative correlation with AP, indicating its predominant role as a mobilized phosphorus source. The progressive reduction in NaOH-P_o_ and HCl-P highlight the compound’s capacity to destabilize recalcitrant organic and mineral-bound phosphorus. These patterns suggest ligand-promoted dissolution of stable P pools, with minimal residual phosphorus variation (<2% change), underscoring the efficacy of trans-aconitic acid in redistributing phosphorus from stable to labile fractions through chemical interaction mechanisms.

In the two contrasting black soils investigated here, citric acid and malic acid emerged as the most effective organic acids for enhancing phosphorus availability, as evidenced by substantial increases in labile P fractions. Tartaric acid showed moderate efficacy in both soils, while trans-aconitic acid exhibited the weakest mobilization capacity in Alfisols. Crucially, the efficacy of these organic acids varied significantly between the Alfisol and Mollisol, with Mollisol demonstrating greater responsiveness in labile P redistribution. This indicates that while root-secreted organic acids can accelerate the transformation of recalcitrant P pools into bioavailable forms, their performance is highly soil-specific. Thus, although organic acids clearly enhance P availability by facilitating legacy P fraction transformation, their practical effectiveness depends critically on inherent soil properties, including mineralogy, organic matter content, pH buffering capacity, and initial P speciation distribution–factors that diverged markedly between the Alfisol and Mollisol studied.

While this study elucidates chemical mechanisms of phosphorus mobilization, several limitations warrant consideration. First, the use of sterilized soils excluded biotic interactions, potentially overestimating direct acid effects; therefore, field validation is required to quantify contributions from microbial P-solubilizing communities. Second, the 60-day incubation captured short-term solubilization dynamics but not long-term P re-fixation processes; studies exceeding 6 months are needed to evaluate the persistence of mobilized P.

## 5 Conclusion

The maize cultivation experiment identified significant accumulation of acetic acid, malic acid, tartaric acid, and trans-aconitic acid in root exudates, with phosphorus limitation inducing marked quantitative changes specifically in malic, citric, tartaric, and trans-aconitic acid levels.

Soil activation studies demonstrated that citric, malic, tartaric, and trans-aconitic acids differentially mobilized residual phosphorus across soil types, targeting distinct P fractions. Citric acid predominantly enhanced labile P by reducing HCl-P, while malic acid concurrently decreased NaOH-P_i_ and HCl-P to achieve similar effects. Tartaric acid exhibited broader activation capacity, reducing NaOH-P_o_, HCl-P, and NaOH-P_i_, whereas trans-aconitic acid uniquely mobilized Res-P in addition to NaOH-P_o_ and HCl-P. pH acidification and ligand exchange mechanisms drove P release in citric and malic acid treatments, while tartaric and trans-aconitic acids additionally promoted organophosphorus mineralization, evidenced by progressive NaOH-P_o_ reduction. These findings establish organic acid-specific pathways for phosphorus mobilization, with HCl-P depletion common to all treatments and NaOH-P_o_/Res-P reduction distinguishing trans-aconitic acid’s activation profile.

## Supporting information

S1 Fig Semi hydroponic cultivation of maize seedlings. (TIF)

S2 Fig Changes in the AP content at various time points in Alfisols amended with LMWOAs over the 60 d incubation period. (LMWOAs applied at 1% by weight in the incubated soil). CK: CK treatment, MA: malic acid, CA: citric acid, TA: tartaric acid, TAa: trans-aconitic acid.
(TIF)

S3 Fig Changes in the AP content at various time points in Alfisols amended with LMWOAs over the 60 d incubation period. (LMWOAs applied at 1.5% by weight in the incubated soil). CK: CK treatment, MA: malic acid, CA: citric acid, TA: tartaric acid, TAa: trans-aconitic acid.
(TIF)

S4 Fig Changes in the AP content at various time points in Mollisols amended with LMWOAs over the 60 d incubation period. (LMWOAs applied at 1% by weight in the incubated soil). CK: CK treatment, MA: malic acid, CA: citric acid, TA: tartaric acid, TAa: trans-aconitic acid.
(TIF)

S5 Fig Changes in the AP content at various time points in Mollisols amended with LMWOAs over the 60 d incubation period. (LMWOAs applied at 1.5% by weight in the incubated soil). CK: CK treatment, MA: malic acid, CA: citric acid, TA: tartaric acid, TAa: trans-aconitic acid.
(TIF)

S1 Table Operating parameters of LC-MSMS. (DOC)

S2 Table Physical and chemical characteristics at various time points in the soils amended with malic acid (malic acid applied at 2% by weight in the incubated soil). (DOC)

S3 Table Physical and chemical characteristics at various time points in the soils amended with tartaric acid (tartaric acid applied at 2% by weight in the incubated soil). (DOC)

S4 Table Physical and chemical characteristics at various time points in the soils amended with citric acid (citric acid applied at 2% by weight in the incubated soil). (DOC)

S5 Table Physical and chemical characteristics at various time points in the soils amended with trans-aconitonic acid (trans-aconitonic acid applied at 2% by weight in the incubated soil). (DOC)
