# Supplementary figures and images for: Organic acid-mediated phosphorus mobilization in black soils: differential effects of maize root exudates on alfisols and mollisols in Northeast China
Source: PLoS One. 2025 Sep 24;20(9):e0333230. doi: 10.1371/journal.pone.0333230 (PMC12459762; doi:10.1371/journal.pone.0333230)

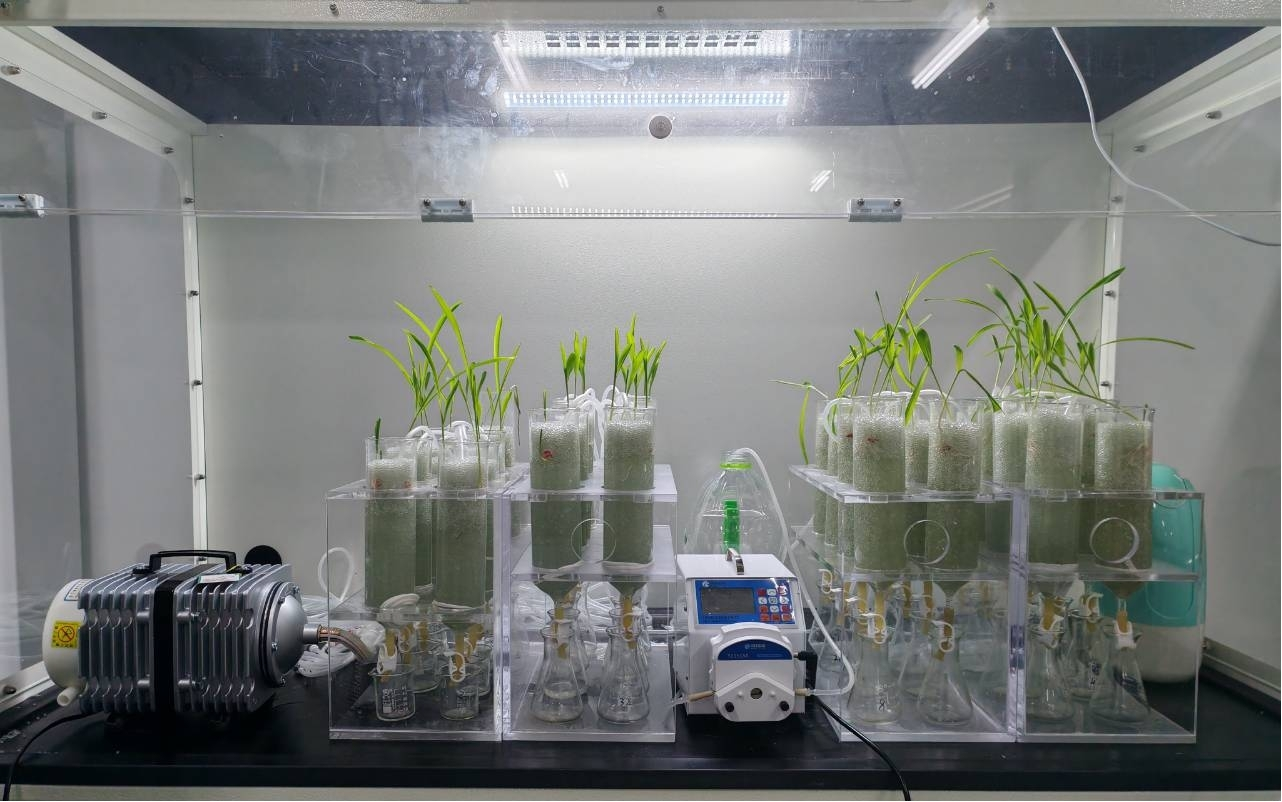

Supplement: S1 Fig — (TIF) [file pone.0333230.s001.tif]

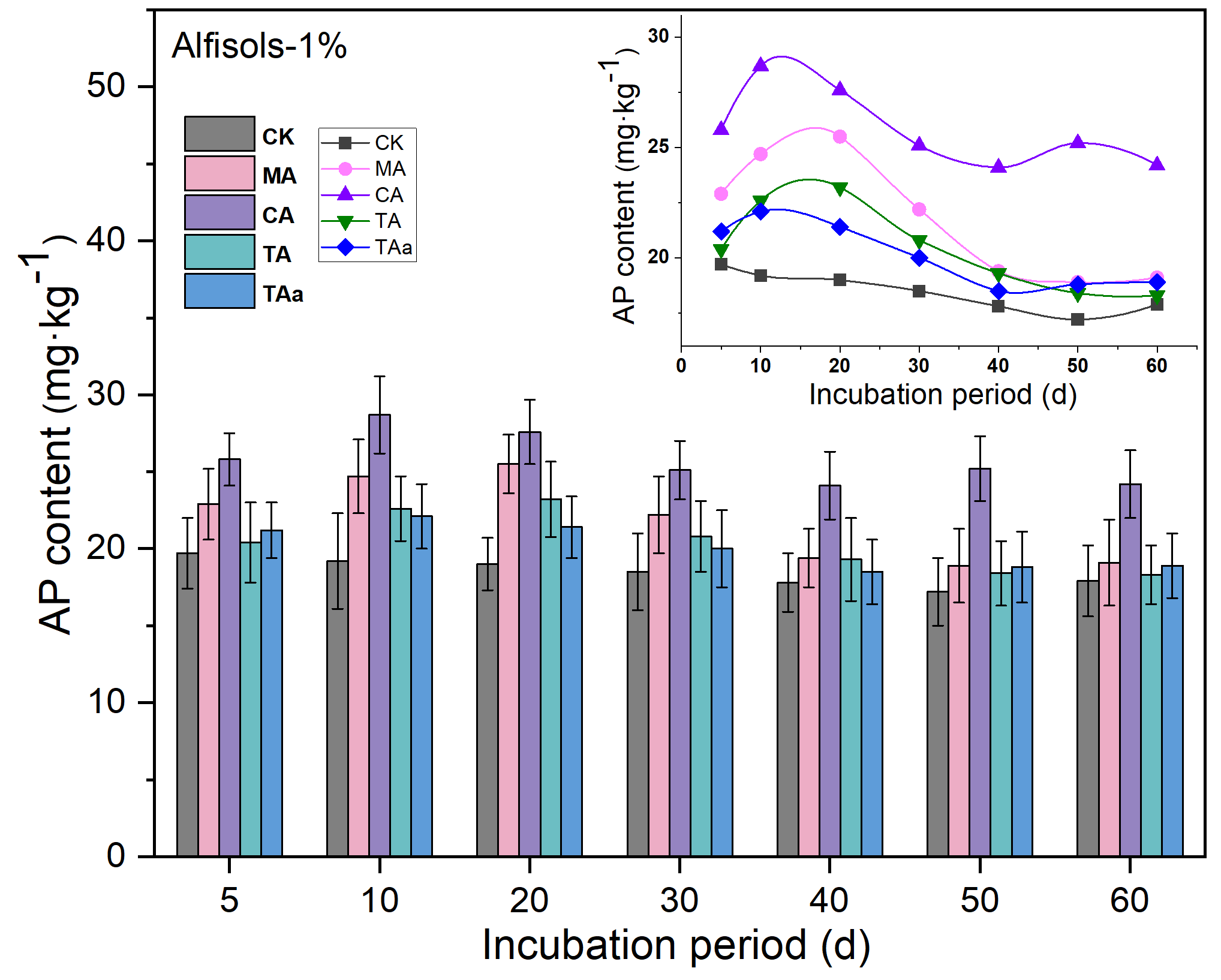

Supplement: S2 Fig — (LMWOAs applied at 1% by weight in the incubated soil). CK: CK treatment, MA: malic acid, CA: citric acid, TA: tartaric acid, TAa: trans-aconitic acid. (TIF) [file pone.0333230.s002.tif]

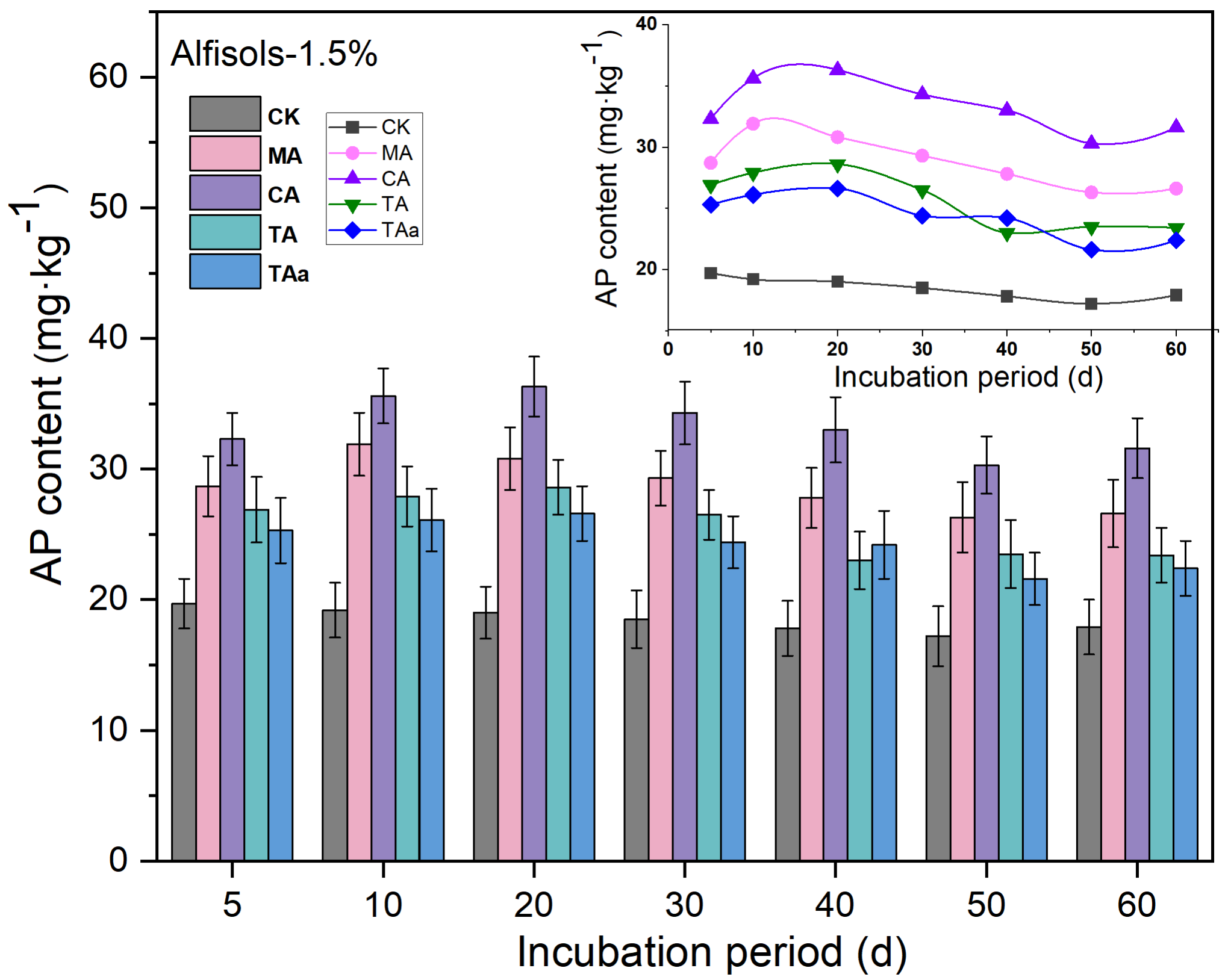

Supplement: S3 Fig — (LMWOAs applied at 1.5% by weight in the incubated soil). CK: CK treatment, MA: malic acid, CA: citric acid, TA: tartaric acid, TAa: trans-aconitic acid. (TIF) [file pone.0333230.s003.tif]

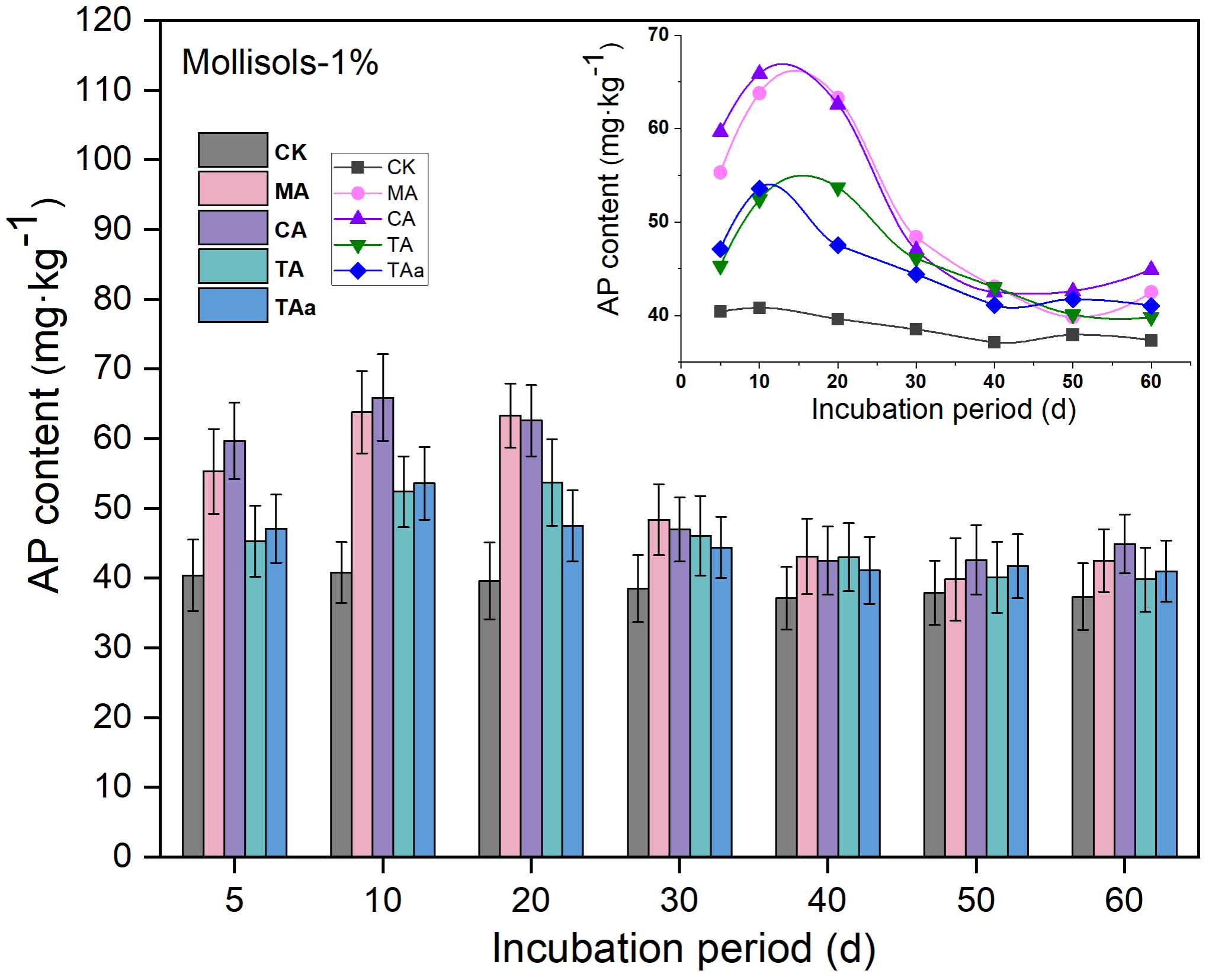

Supplement: S4 Fig — (LMWOAs applied at 1% by weight in the incubated soil). CK: CK treatment, MA: malic acid, CA: citric acid, TA: tartaric acid, TAa: trans-aconitic acid. (TIF) [file pone.0333230.s004.tif]

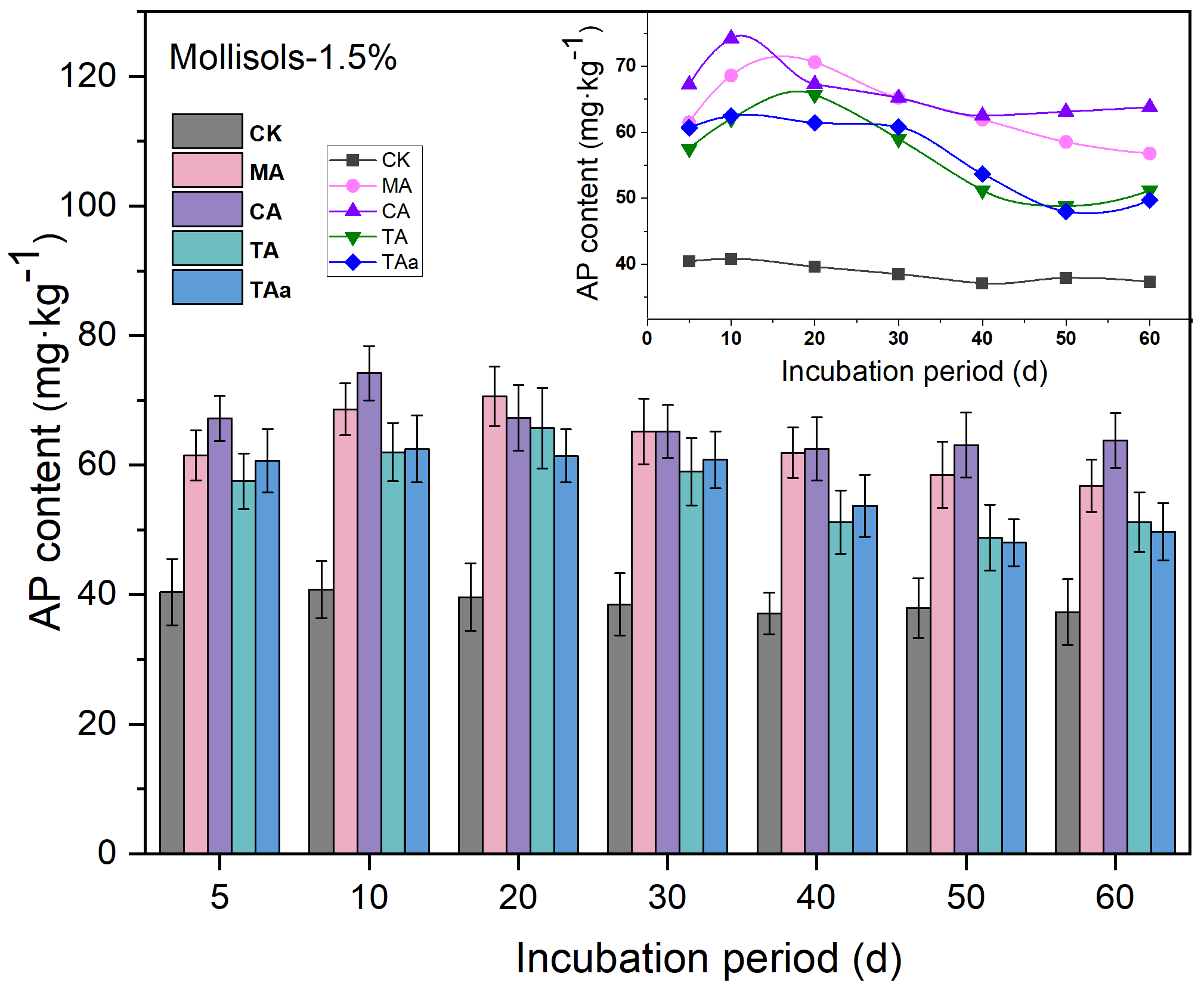

Supplement: S5 Fig — (LMWOAs applied at 1.5% by weight in the incubated soil). CK: CK treatment, MA: malic acid, CA: citric acid, TA: tartaric acid, TAa: trans-aconitic acid. (TIF) [file pone.0333230.s005.tif]
