# Supplementary material for: Organic acid-mediated phosphorus mobilization in black soils: differential effects of maize root exudates on alfisols and mollisols in Northeast China
Source: PLoS One. 2025 Sep 24;20(9):e0333230. doi: 10.1371/journal.pone.0333230 (PMC12459762; doi:10.1371/journal.pone.0333230)
Supplement: S1 Table — (DOC) [file pone.0333230.s006.doc]

Table S1 Operating parameters of LC-MSMS

| Item | Parameter |
| --- | --- |
| Chromatographic Column | Shim-pack GIS-HP C18-AQ（2.1 mm I.D.×100 mm L,3 μm） |
| Mobile Phase | Phase A: 0.1% formic acid aqueous solution; Phase B: Methanol  Phase B: Methanol  Phase A: 0.1% formic acid aqueous solution  Phase B: Methanol  Phase A: 0.1% formic acid aqueous solution  Phase B: Methanol |
| Flow Rate | 0.2 mL/min |
| Injection Volume | 2 μL |
| Column Temperature | 35 °C |
| Elution Mode Gradient elution | Gradient elution |
| Initial Composition | Phase A: 95%; Phase B: 5%  Phase A: 95% |
| Ion Source | ESI(-) |
| Interface Voltage | -4.5 kv |
| Nebulizer Flow Rate | 3 L/min |
| Heating Block Temperature | 400 °C |
| Heating Gas Flow Rate | 10.0 L/min |
| DL Temperature | 250 °C |
| Interface Temperature | 300 °C |
| Drying Gas Flow Rate | 10.0 L/min |
| Scan Mode | Multiple Reaction Monitoring (MRM) |
