# Supplementary material for: Organic acid-mediated phosphorus mobilization in black soils: differential effects of maize root exudates on alfisols and mollisols in Northeast China
Source: PLoS One. 2025 Sep 24;20(9):e0333230. doi: 10.1371/journal.pone.0333230 (PMC12459762; doi:10.1371/journal.pone.0333230)
Supplement: S2 Table — (DOC) [file pone.0333230.s007.doc]

**Table S2** Physical and chemical characteristics at various time points in the soils amended with malic acid (malic acid applied at 2% by weight in the incubated soil)

| Incubation period (d) | **pH** | | **Corg (g kg^-1^)** | | **DOC (g kg^-1^)** | | **Alkeline-N (mg kg^-1^)** | | **CEC (mg kg^-1^)** | | **TP (mg kg^-1^)** | |
| --- | --- | --- | --- | --- | --- | --- | --- | --- | --- | --- | --- | --- |
|  | Alfisols | Mollisols | Alfisols | Mollisols | Alfisols | Mollisols | Alfisols | Mollisols | Alfisols | Mollisols | Alfisols | Mollisols |
| CK | 6.67 | 5.66 | 37.21 | 33.36 | 0.27 | 0.23 | 110.7 | 233.1 | 25.4 | 26.9 | 569 | 672 |
| 5 | 5.73 | 4.59 | 40.29 | 37.11 | 0.53 | 0.48 | 119.1 | 239.9 | 25.7 | 27.0 | 573.2 | 669.7 |
| 10 | 6.42 | 4.91 | 40.29 | 36.92 | 0.32 | 0.26 | 140.4 | 277.8 | 25.7 | 24.9 | 569.9 | 670.5 |
| 20 | 6.61 | 5.52 | 40.61 | 36.88 | 0.35 | 0.26 | 124.8 | 257.9 | 25.1 | 28.0 | 583.0 | 673.1 |
| 30 | 6.69 | 5.69 | 39.85 | 35.71 | 0.31 | 0.27 | 126.3 | 248.6 | 25.3 | 27.3 | 576.9 | 677.9 |
| 40 | 6.73 | 5.77 | 40.07 | 34.99 | 0.33 | 0.26 | 117.6 | 236.8 | 25.1 | 26.3 | 569.0 | 665.4 |
| 50 | 6.82 | 5.74 | 40.17 | 35.25 | 0.34 | 0.25 | 116.7 | 241.2 | 23.7 | 25.8 | 568.5 | 669.9 |
| 60 | 6.84 | 5.87 | 40.15 | 35.31 | 0.31 | 0.24 | 115.7 | 239.0 | 26.2 | 25.8 | 570.4 | 667.8 |
