# Supplementary material for: Organic acid-mediated phosphorus mobilization in black soils: differential effects of maize root exudates on alfisols and mollisols in Northeast China
Source: PLoS One. 2025 Sep 24;20(9):e0333230. doi: 10.1371/journal.pone.0333230 (PMC12459762; doi:10.1371/journal.pone.0333230)
Supplement: S3 Table — (DOC) [file pone.0333230.s008.doc]

**Table S3** Physical and chemical characteristics at various time points in the soils amended with tartaric acid (tartaric acid applied at 2% by weight in the incubated soil)

| Incubation period (d) | **pH** | | **Corg (g kg^-1^)** | | **DOC (g kg^-1^)** | | **Alkeline-N (mg kg^-1^)** | | **CEC (mg kg^-1^)** | | **TP (mg kg^-1^)** | |
| --- | --- | --- | --- | --- | --- | --- | --- | --- | --- | --- | --- | --- |
|  | Alfisols | Mollisols | Alfisols | Mollisols | Alfisols | Mollisols | Alfisols | Mollisols | Alfisols | Mollisols | Alfisols | Mollisols |
| CK | 6.67 | 5.66 | 37.21 | 33.36 | 0.27 | 0.23 | 110.7 | 233.1 | 25.4 | 26.9 | 569 | 672 |
| 5 | 5.82 | 4.73 | 42.15 | 38.77 | 0.71 | 0.66 | 112.6 | 236.8 | 25.6 | 27.1 | 567.6 | 676.3 |
| 10 | 6.53 | 5.08 | 42.08 | 38.58 | 0.52 | 0.45 | 130.0 | 249.9 | 26.2 | 26.6 | 568.1 | 679.3 |
| 20 | 6.81 | 5.63 | 42.17 | 38.64 | 0.48 | 0.41 | 117.6 | 255.2 | 24.7 | 26.9 | 562.1 | 683.9 |
| 30 | 6.89 | 5.71 | 41.81 | 37.47 | 0.47 | 0.41 | 119.7 | 244.6 | 25.2 | 26.2 | 568.1 | 674.5 |
| 40 | 6.93 | 5.84 | 41.83 | 37.75 | 0.47 | 0.39 | 115.4 | 239.3 | 24.7 | 26.6 | 568.1 | 679.1 |
| 50 | 6.92 | 5.88 | 41.93 | 37.11 | 0.48 | 0.40 | 115.5 | 231.8 | 24 | 26.8 | 580.1 | 685.0 |
| 60 | 6.99 | 5.96 | 41.91 | 37.37 | 0.46 | 0.41 | 113.2 | 234.9 | 24.4 | 27.3 | 560.3 | 676.1 |
