# Supplementary material for: Organic acid-mediated phosphorus mobilization in black soils: differential effects of maize root exudates on alfisols and mollisols in Northeast China
Source: PLoS One. 2025 Sep 24;20(9):e0333230. doi: 10.1371/journal.pone.0333230 (PMC12459762; doi:10.1371/journal.pone.0333230)
Supplement: S4 Table — (DOC) [file pone.0333230.s009.doc]

**Table S4** Physical and chemical characteristics at various time points in the soils amended with citric acid (citric acid applied at 2% by weight in the incubated soil)

| Incubation period (d) | **pH** | | **Corg (g kg^-1^)** | | **DOC (g kg^-1^)** | | **Alkeline-N (mg kg^-1^)** | | **CEC (mg kg^-1^)** | | **TP (mg kg^-1^)** | |
| --- | --- | --- | --- | --- | --- | --- | --- | --- | --- | --- | --- | --- |
|  | Alfisols | Mollisols | Alfisols | Mollisols | Alfisols | Mollisols | Alfisols | Mollisols | Alfisols | Mollisols | Alfisols | Mollisols |
| CK | 6.67 | 5.66 | 37.21 | 33.36 | 0.27 | 0.23 | 110.7 | 233.1 | 25.4 | 26.9 | 569.0 | 672 |
| 5 | 5.88 | 4.61 | 42.42 | 39.21 | 0.61 | 0.63 | 114.6 | 240.8 | 23.8 | 26.8 | 573.4 | 668.1 |
| 10 | 6.63 | 4.92 | 42.44 | 39.25 | 0.42 | 0.42 | 133.9 | 261.5 | 25.4 | 27.9 | 574.2 | 669.8 |
| 20 | 6.84 | 5.56 | 42.12 | 38.91 | 0.43 | 0.4 | 134.2 | 269.9 | 26.4 | 27.1 | 573.4 | 678.3 |
| 30 | 6.89 | 5.84 | 42.38 | 38.74 | 0.38 | 0.39 | 123.2 | 252.4 | 25 | 26.7 | 572.9 | 666.4 |
| 40 | 7.14 | 5.88 | 42.16 | 38.02 | 0.39 | 0.41 | 117.9 | 240.5 | 25.7 | 26.8 | 573.3 | 681.7 |
| 50 | 7.14 | 5.97 | 42.25 | 37.28 | 0.37 | 0.40 | 116.9 | 238.9 | 25.6 | 27.4 | 575.3 | 677.1 |
| 60 | 7.22 | 6.02 | 42.18 | 37.34 | 0.37 | 0.41 | 117.9 | 238.2 | 24.5 | 27.5 | 570.7 | 681.7 |
