# Supplementary material for: Organic acid-mediated phosphorus mobilization in black soils: differential effects of maize root exudates on alfisols and mollisols in Northeast China
Source: PLoS One. 2025 Sep 24;20(9):e0333230. doi: 10.1371/journal.pone.0333230 (PMC12459762; doi:10.1371/journal.pone.0333230)
Supplement: S5 Table — (DOC) [file pone.0333230.s010.doc]

**Table S5** Physical and chemical characteristics at various time points in the soils amended with trans-aconitonic acid (trans-aconitonic acid applied at 2% by weight in the incubated soil)

| Incubation period (d) | **pH** | | **Corg (g kg^-1^)** | | **DOC (g kg^-1^)** | | **Alkeline-N (mg kg^-1^)** | | **CEC (mg kg^-1^)** | | **TP (mg kg^-1^)** | |
| --- | --- | --- | --- | --- | --- | --- | --- | --- | --- | --- | --- | --- |
|  | Alfisols | Mollisols | Alfisols | Mollisols | Alfisols | Mollisols | Alfisols | Mollisols | Alfisols | Mollisols | Alfisols | Mollisols |
| CK | 6.67 | 5.66 | 37.21 | 33.36 | 0.27 | 0.23 | 110.7 | 233.1 | 25.4 | 26.9 | 569.0 | 672.0 |
| 5 | 5.85 | 4.86 | 42.55 | 39.62 | 0.88 | 0.91 | 112.0 | 242.9 | 25.5 | 26.5 | 572.1 | 668.8 |
| 10 | 6.53 | 5.26 | 42.64 | 39.65 | 0.75 | 0.84 | 115.6 | 250.3 | 26.1 | 26.5 | 561.5 | 683.6 |
| 20 | 6.71 | 5.72 | 42.62 | 39.41 | 0.74 | 0.72 | 119.5 | 238.6 | 25.8 | 26.8 | 577.7 | 667.9 |
| 30 | 6.84 | 5.79 | 42.38 | 39.24 | 0.75 | 0.68 | 112.0 | 235.8 | 24.7 | 27.2 | 566.4 | 677.7 |
| 40 | 6.81 | 5.87 | 42.26 | 38.52 | 0.74 | 0.69 | 113.8 | 233.5 | 25.8 | 26.9 | 579.0 | 671.6 |
| 50 | 6.88 | 5.94 | 41.75 | 38.28 | 0.73 | 0.69 | 112.7 | 236.5 | 25.6 | 27.9 | 563.9 | 669.5 |
| 60 | 6.91 | 6.18 | 41.68 | 37.84 | 0.73 | 0.70 | 114.2 | 235.3 | 24.1 | 26.8 | 566.7 | 675.3 |
